# Case Report: PANDAS and Persistent Lyme Disease With Neuropsychiatric Symptoms: Treatment, Resolution, and Recovery

**DOI:** 10.3389/fpsyt.2021.505941

**Published:** 2021-02-02

**Authors:** Amy Cross, Denis Bouboulis, Craig Shimasaki, Charles Ray Jones

**Affiliations:** ^1^Moleculera Labs, Oklahoma City, OK, United States; ^2^Advanced Allergy, Immunology and Asthma, Darien, CT, United States; ^3^Private Medical Practice, New Haven, CT, United States

**Keywords:** Lyme, PANDAS, Cunningham Panel, neuropsychiatric, IVIg, strep pharyngitis, basal ganglia encephalitis (BGE), autoimmune encephalitis

## Abstract

This case report describes the diagnosis and treatment of a pre-pubertal (onset at age 7) Caucasian female with serological evidence of Lyme disease accompanied by multiple neuropsychiatric symptoms 6 months following a vacation in a tick endemic area of the United States. Prior to the diagnosis of Lyme disease, the patient also met the clinical diagnostic criteria for PANDAS (Pediatric Autoimmune Neuropsychiatric Disorder Associated with Strep), with serological evidence of three distinct episodes of streptococcal pharyngitis. All three episodes of strep occurred during the 6-months interval between suspected Lyme disease exposure and the onset of multiple neuropsychiatric symptoms. Her sometimes incapacitating symptoms followed a relapsing and remitting course that impacted her personal, family, social, and academic domains. Over a span of 31 consecutive months of treatment with various antimicrobials and three courses of intravenous immunoglobulins (IVIg) she experienced complete remission and remains symptom free at the time of this publication. Written permission was obtained from the minor patient's mother allowing the submission and publication of this case study.

## Background

In current medical practice, patients with co-occurring Lyme borreliosis and autoimmune encephalitidies secondary to strep infections, such as PANDAS (1–3), are met with a number of inherent challenges (4). The ability to obtain accurate serological testing results for Lyme disease and common co-infections is a challenge for patients and providers alike due to the varied reported accuracy of different Lyme tests (5). To complicate things further, Lyme disease testing is fraught with controversy regarding methodology and interpretation of test results. In 1980, the Centers for Disease Control and Prevention (CDC) began surveillance for Lyme disease, identifying only 10 states where Lyme disease was believed to occur. Currently, all 50 states have reported cases of Lyme disease (6). In 2017, the CDC received reports of a total of 42,743 confirmed and probable cases of Lyme disease, but they estimate that in the US ~300,000 patients may contract Lyme disease annually (7). One clinical sign of Lyme disease exposure from a tick bite is an erythema migrans (EM) rash, but often patients with documented Lyme disease do not present with EM (8, 9).

Medical literature supports numerous cases of neuropsychiatric symptoms in children who have a diagnosis of Lyme disease as well as other tick-borne infections. For example, a 14-year-old boy experienced a sudden onset of psychotic behavior which persisted despite multiple hospitalizations and treatment with psychotropic medications. He was subsequently diagnosed with neurobartonellosis after he developed cutaneous lesions, which has been documented as common in individuals reporting neuropsychiatric symptoms and *Bartonella* spp. infection or exposure (10). He was treated with a combination of antimicrobials and experienced a gradual progressive decrease in neuropsychiatric symptoms and was able to discontinue all psychotropic drugs (11). Another well-documented case describes a 12-year-old boy who had a compulsion to pedal a stationary bicycle, unwilling to stop long enough to eat or go to school, resulting in a 30-pound weight loss, a skeletal appearance, and multiple hospitalizations. He was found to be infected with Borrelia and recovered after a course of intravenous penicillin (12).

An extensive review article documents increasing evidence and recognition that Lyme borreliosis can cause psychiatric symptoms (13). Drawing from databases and using search engines along with clinical experiences, the authors concluded that Borrelia can “cause immune and metabolic effects that result in a gradually developing spectrum of neuropsychiatric symptoms usually presenting with significant comorbidity which may include developmental disorders, autism spectrum disorders, schizoaffective disorders, bipolar disorder, depression, anxiety disorders (panic disorder, social anxiety disorder, generalized anxiety disorder, posttraumatic stress disorder, and intrusive symptoms), eating disorder, decreased libido, sleep disorder, addiction, opioid addiction, cognitive impairments, dementia, seizure disorders, suicide, violence, anhedonia, depersonalization, dissociative episodes, derealization, and other impairments" (13).

Data from an unpublished survey of over 1,000 parents of children with PANDAS and/or PANS, conducted by Moleculera Labs in 2018, “Economic and Psychosocial Costs of PANDAS and PANS,” revealed that, on average, patients have seen up to 12 medical providers, requiring ~3 years before receiving a diagnosis of PANDAS or its broader diagnostic category, PANS (Pediatric Acute-onset Neuropsychiatric Syndrome). The survey results also revealed that at least 20% of patients with PANDAS and/or PANS experience a delay of more than 12 months before receiving appropriate treatment even after being diagnosed with this type of autoimmune encephalopathy.

## Introduction

This is a case report of a previously healthy 7-year-old girl who was conceived through artificial insemination resulting in an uncomplicated, full-term pregnancy and delivery by caesarian section. She weighed 8 pounds, 10 ounces at birth and was breast fed. Her family history was unremarkable for rheumatic fever, tics, OCD, autoimmune disorders, psychiatric illness, or allergies. 6 months prior to the onset of her symptoms, the patient and her family vacationed in a tick endemic area of the US, but the patient had no known tick attachment or erythema migrans (EM) rash. Quite abruptly, over a period of 3 weeks, the patient experienced dramatic declines in cognitive functioning, concentration, and ability to focus, a loss of math skills, the onset of dysgraphia and difficulty with social cues, decreased processing speed, word selection problems, anxiety, fatigue, nighttime awakening, chills, joint and muscle pain, moodiness, both general and separation anxiety, and panic attacks. She also experienced obsessions and compulsions and aggressive behavior which was completely out of character according to her mother. She said to her mother, “Mom, something happened to my brain.” Previously, she enjoyed music and dance lessons but her overall activity was decreased. Because the patient had consistently been identified as an academically gifted child, a call from the patient's teacher came as a surprise when the mother was asked, “Did you drop your daughter on her head?” The patient regressed from being a year ahead of her class in math, to being unable to add beyond the number 10. She began having trouble comprehending more difficult reading. During a ride home with her mother, the patient asked, “Who are you? What's your name again?” And “I know you are mommy but what's your name?” Her mother began to think that her daughter may have experienced an accident or head injury, but there was no physical sign or history of any type of injury. An EEG was performed with normal findings.

## Case Presentation

**Figure 1:**
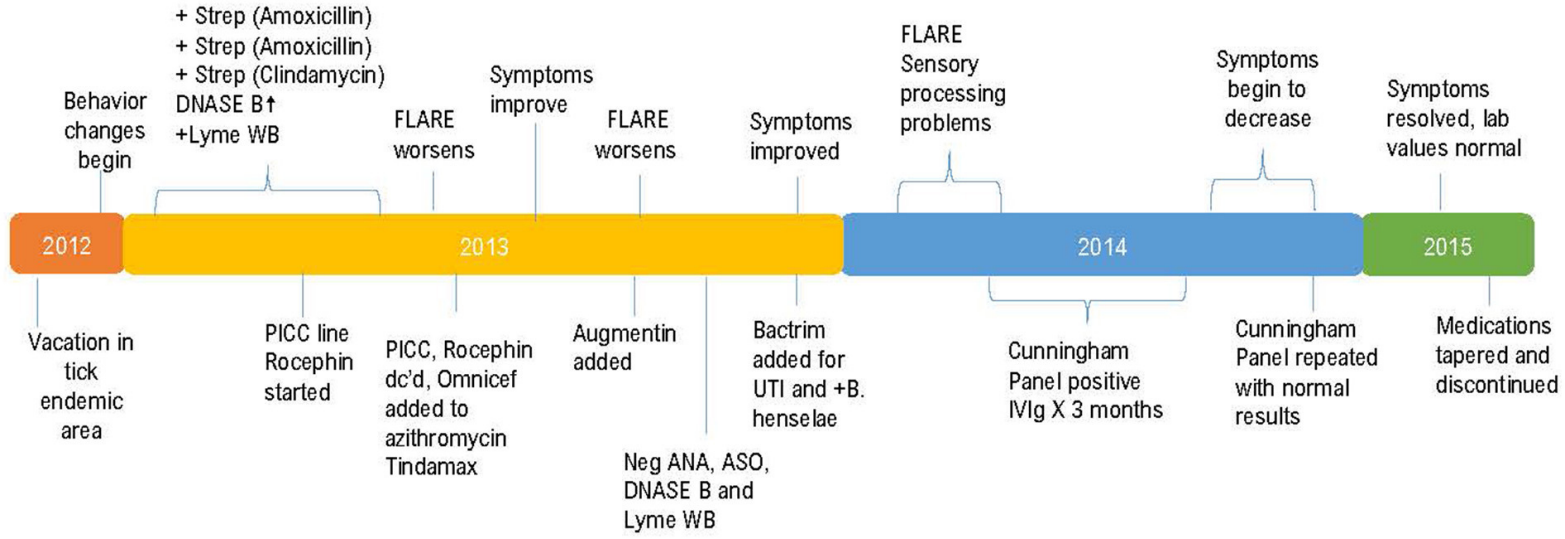
Timeline between probable disease exposure and completion of treatment.

This previously asymptomatic, healthy 7-year-old girl experienced an abrupt onset of several physical, neurological, and psychiatric symptoms increasing in intensity over a 3-week period. The patient's mother reports that strep pharyngitis was diagnosed by a previous physician on three separate occasions with the first episode occurring 180 days prior to the onset of neuropsychiatric symptoms. The first course of treatment was a 10-day course of amoxicillin which resulted in no change in her behavior or functioning. The strep infection recurred, and amoxicillin was again prescribed with no behavioral improvement. The third episode of strep throat (Quest Labs DNase B results of 407), (reference range <376) was treated with a course of clindamycin with a notable improvement in the patient's symptoms (14). Neurologists at a university medical center referred the patient to the Psychiatry department. Instead, the patient's mother chose to have her evaluated by a developmental pediatrician who arrived at a diagnosis of Pediatric Autoimmune Neuropsychiatric Disorder Associated with Strep (PANDAS) (15, 16). Due to the patient's worsening symptoms, a second opinion was sought, and additional blood work performed including a CDC Lyme Western Blot which was positive, leading to the additional diagnosis of Lyme disease. Quest Laboratories' Lyme disease enzyme immunoassay (EIA) with reflex to Western Blot was positive at 1.25, (0.00–0.90 negative index value). The patient was referred to the university's infectious disease clinic where treatment to address the Lyme disease diagnosis began. A PICC line was inserted to facilitate the administration of IV Rocephin. A lumbar puncture (LP) was performed as part of the infectious disease evaluation with normal results. At this point, because the patient had both a diagnosis of PANDAS and Lyme disease, her mother sought the opinion of a pediatric Lyme disease specialist who was also familiar with diagnosing and treating PANDAS. A graphic representation of the timeline from the initial suspected Lyme disease exposure through her complete course of treatment is shown in Figure 1.

On her first visit with the pediatric Lyme disease specialist, the patient presented with crying, anxiety, headache, joint pain, decreased cognitive functioning, fatigue, nighttime awakening and an extreme fear of sleeping alone. Her ASO titer was negative but a DNase B titer was elevated at 407 (<376). Her ESR was normal. The IV Rocephin 1.5 gm QD prescribed by the previous doctor was continued, but her PICC line became occluded requiring removal. Oral Omnicef 300 mg BID was initiated to replace the Rocephin. Zithromax 250 mg BID and Tindamax 250 mg QD (Saturdays and Sundays only) were initiated.

Due to complaints of right upper quadrant pain, an abdominal ultrasound was ordered which was unremarkable ruling out concerns of possible Rocephin cholelithiasis. Ursodiol 300 mg BID was ordered to help break down cholesterol that had possibly formed in the gall bladder. A CDC Lyme WB was negative although repeated Lyme testing through IGeneX Laboratory revealed a positive Lyme WB IgM with positive double starred bands 31 and 41 and indeterminate bands 39 and 83–93. Lyme WB IgG and *Bartonella henselae* testing showed negative IgG and IgM results. Babesia FISH (RNA) was negative. Her previously elevated DNase B was now normal as was her ASO titer. An elevated *Mycoplasma pneumoniae* IgG at 2.01 (reference range ≤ 0.90 ISR) indicated a previous infection and *M. pneumoniae* IgM was negative. A basic metabolic panel, CBC, urinalysis, ESR, and ANA were completely within normal limits. Genetic testing indicated that the patient was a carrier of the *HLA-DRB1, 2*, and *4* genes which are reported to occur more frequently in patients with Lyme disease and rheumatoid arthritis (17–19). It is also been observed that *B. burgdorferi* can alter the repertoire of self-peptides bound to MHC class II molecules and influence the likelihood of autoreactive T-cells which could lead to infection-induced autoimmune illnesses (20).

The patient's mother was given a Lyme disease checklist to complete prior to each appointment indicating symptoms and improvement or lack of improvement since the previous visit. The Lyme disease checklist asks the parent to rate the patient on 35 symptoms including (1) unexplained fevers, sweats, chills or flushing, (2) weight change (loss or gain), (3) fatigue, (4) hair loss, (5) swollen glands, (6) sore throat, (7) testicular/pelvic pain, (8) menstrual irregularity, (9) unexplained milk production/breast pain, (10) irritable bladder or dysfunction, (11) upset stomach/abdominal pain, (12) constipation/diarrhea, (13) chest pain/rib soreness, (14) shortness of breath or cough, (15) heart palpitations, (16) joint pain/swelling, (17) joint stiffness, (18) muscle pain/cramps, (19) muscle twitching, (20) headaches, (21) neck creaks/cracks/stiffness, (22) numbness/tingling/stabbing sensations, (23) facial paralysis, (24) double vision/floaters/loss of vision, (25) buzzing/ringing/ear pain/sensitivity, (26) vertigo/dizziness, (27) lightheadedness/poor balance, (28) tremors, (29) difficulty thinking, (30) difficulty concentrating, (31) forgetfulness/poor short term memory, (32) disorientation/getting lost, (33) difficulty with speech or writing, (34) mood swings/irritability/depression, and (35) too much/too little sleep. In addition, the parents were asked to rate symptom severity on a mild, moderate, or severe scale as well as symptom frequency on an occasional, often, or constant scale.

At a follow-up office visit 8 weeks later, her Lyme disease checklist indicated overall improvement with no headaches, but increased insomnia and scattered joint sensitivities. Range of motion, biometrics and vital signs were normal except for an oral temperature of 99.2° F. She had no “dark circles” under her eyes, no tremors and her balance was normal. 2 weeks later the patient experienced a sudden symptom recurrence. Omnicef was discontinued, Zithromax continued at the same dosage and Augmentin added at 500 mg BID. EMLA cream (Lidocaine 2.5% and Prilocaine 2.5%) was prescribed as a topical anesthetic to treat her joint sensitivities. Lab values at this visit were within normal limits.

10 weeks following the regression, at her next office visit, she had no major complaints and her Lyme disease checklist indicated overall symptom improvement. She denied having headaches and reported increased energy, improved cognitive function and enjoyment of her music lessons. Her physical examination was within normal limits. ANA, CRP, ASO, Streptozyme, and *M. pneumoniae* lab results were all within normal limits. The patient was attending day camp, Monday through Friday, all day, without problems. 2 weeks following this appointment, she suddenly became argumentative, hyperactive, and combative and experienced chills and headache. Bactrim SS BID was added due to a suspected urinary tract infection and to address new IGeneX laboratory results indicating the presence of Lyme borreliosis with positive double starred bands 31 and 41 and double starred bands 39, 83–93 indeterminate. Quest laboratory testing showed an elevated *B. henselae* IgG at 1:64. *B. henselae* IgM was within normal limits at <1:20. Both Ehrlichia and Anaplasma testing had negative results.

2 months later at her next office visit, the patient presented as unhappy and experiencing some facial “twitching.” However, her appetite was good (no previous restrictive eating or food refusal was noted) and she was engaged in activities including dance and tennis.

Two additional months later, labs were repeated. Quest laboratory testing indicated negative results for *E. chaffeensis, A. phagocytophilum*, and *B. henselae* IgG and IgM. IGeneX testing showed an indeterminate Lyme IgM with double starred bands 31 and 41. Lyme IgG testing was interpreted as positive with double starred bands 31, 41, and 58 reactive. IGeneX interpretation guidance states the IgG WB is considered positive if two or more of the double starred bands are present from either Group 1 or Group 2. Group 1 includes bands 23–25, 31, 34, 39, 83–93. Group 2 includes bands 23–25, 34, 39, 41, 83–93. *B. henselae* IFA IgG and IgM were both reported as negative. *B. duncani* IgG was negative but IgM was reported as indicative of active infection at a level of 80 with values > 40 considered elevated. This was presumed to be a co-infection due to the original tick exposure as no additional tick exposure was identified.

CD45RA testing, which is a marker of naïve T cells, showed an elevated result of 38% (reference range 5–37%) which is an indication of the amount of time elapsed (~10 days) since the most recent antigenic stimulation (21, 22). Although the patient's CD45RA was not significantly abnormal, as it was only slightly outside of the upper end of the normal range, it may be supportive of the confirmed presence of an additional co-infection with *B. duncani*. The presence of *B. duncani* prompted the addition of Mepron 375 mg BID to her medication regimen. Less than 5 days following this visit, she experienced an onset of severe stomach aches and “migraines” which caused her to leave school early 4 out of 5 days each week for several weeks. These symptoms slowly resolved, and her medication regimen was continued without additional changes.

4 months later at her sixth office visit, her mother reported that she was having panic attacks and was “terrified” to sleep alone. She had some residual combativeness, lack of focus, and cognitive interference. The patient's energy level was improved, and she was engaging in her normal activities. Physical examination and vital signs were normal. Sensory processing problems were evident with heightened sensitivity to lights and sounds. She appeared to manage well at home but struggled with these symptoms in her classroom resulting in the development and implementation of an Individual Educational Plan (IEP). Neuropsychiatric testing was ordered to assess this issue. Quantitative immunoglobulin values were all within normal limits. Quest laboratory testing at this visit revealed negative results for *B. henselae* IgG and IgM, *E. chaffeensis* IgG and IgM, and *Anaplasma Phagocytophilum* IgG and IgM. IGeneX testing showed negative results for *B. henselae* IgG and IgM, and Lyme WB IgG. Lyme WB IgM was indeterminate.

The Cunningham Panel™ of Tests (Moleculera Labs, Oklahoma City, OK) was ordered to assess the presence of antineuronal antibodies against specific neuronal receptors. The patient's Anti-Dopamine D1 Receptor (DRD1) and Anti-Dopamine D2L Receptor (DRD2L) were elevated, her Anti-Lysoganglioside-GM1 (LYSO-GM1) was normal, her Anti-Tubulin (TUB) was elevated, and her Calcium/calmodulin-dependent protein kinase II (CaMKII) was normal (23–25). Published studies demonstrated that the elevated presence of one or more of these antineuronal antibodies and antibody mediated stimulation of CamKII was strongly associated with autoimmune neuropsychiatric symptoms such as those present in PANDAS and PANS (26, 27). Based upon the patient's Cunningham Panel™ test results (See Table 1) the decision was made to prescribe intravenous immunoglobulin (IVIg) in accordance with established treatment guidelines for the patient's level of symptom severity (28, 29). She received IVIg at 2 gm/kg given over a 2-days period for a total of three treatments at 4-weeks intervals.

**Table 1:** Cunningham Panel results—test 1.

	**Dopamine D1 (titer)**	**Dopamine D2 (titer)**	**Lysoganglioside (titer)**	**Tubulin (titer)**	**CaM Kinase II (% of baseline)**
Patient result	8,000	>32,000	320	4,000	119
Normal ranges	500–2,000	2,000–8,000	80–320	250–1,000	53–130
Normal mean	1,056	6,000	147	609	95
Interpretation	Elevated	Elevated	Borderline	Elevated	Normal

A significant evidence base exists for the use of IVIg in PANDAS patients, particularly those exhibiting a relapsing and remitting course of illness with moderate to severe symptoms, although additional randomized, double blind, placebo control studies need to be performed (28). One well-known study, based on the hypothesis that PANDAS and Sydenham's chorea have similar group A strep etiology, proposed that immunomodulatory treatments could effectively treat neuropsychiatric symptoms. Comparing IVIg to therapeutic plasma exchange (TPE) showed that reassessment at 1-month following treatment, the TPE and IVIg groups both showed “striking improvements in obsessive-compulsive symptoms, anxiety, depression, emotional lability, and global functioning" (30).

Although controlled trials have only evaluated IVIg given as a single course, unpublished data based on the experiences of clinicians and researchers engaged in the PANS Consortium suggest that one to three repeated doses of IVIg may be helpful in children who exhibit a positive response to the initial dose but then experience a relapse as the exogenous antibodies are cleared (28).

In another randomized, controlled trial of IVIg for PANDAS, it was concluded that IVIg was safe and well-tolerated but differences between groups were smaller than anticipated and the double-blind comparison failed to demonstrate superiority of IVIg over placebo (31). It was proposed that the study design may have negated the potential observation of an improvement with IVIg by the administration of antibiotics to both groups prior to either IVIg treatment or placebo. An additional study concluded that children with PANDAS derive a favorable response to IVIg at 12-months follow up “consistent with its role in Ig replacement and immune modulation" (32).

At the conclusion of her IVIg treatments, the patient, now age 9 and in third grade, was normal in height and weight. She had no complaints and her activity level continued to improve with decreased fatigue. Her appetite was normal, and her Lyme disease checklist indicated overall improvement. She did have some residual obsessions and compulsions, intermittent hand tingling, slight facial tics, and a humming tic. Medications at this time included Mepron 375 mg BID, Omnicef 300 mg BID, Tindamax 250 mg QD (Saturdays and Sundays only), and Zithromax 250 mg QD, all of which were continued due to the presence of her residual symptoms. The protocol utilized regarding the length of time for which antibiotic treatments were administered was based upon on the patient's symptoms and laboratory results. Before discontinuance of antibiotic treatment, the patient must exhibit symptom resolution along with negative laboratory findings for a at least 2 consecutive months.

Now 21 months into her treatment, at her seventh office visit, her mother reported that she was doing “pretty well” despite a symptom flare the previous month which lasted ~3 weeks but had completely resolved. Her Lyme disease checklist indicated that her status was stable. Vital signs were normal except for a slight elevation in her oral temperature at 99.3 F. The patient was doing well in her new school, had a normal activity level and appetite, and was enjoying her usual activities including music and dance lessons. Physical exam revealed no involuntary movements, no vocal or motor tics, clear lungs, and normal reflexes. IGeneX testing at this time showed indeterminate results for Lyme WB IgM with double starred bands 31 and 41 ++ and double starred bands 39 and 83–93 indeterminate. Lyme WB IgG was negative. *B. henselae* IFA IgG and IgM were negative. *B. duncani* IFA IgG was negative, however, *B. duncani* IgM was positive at 80 with normal values falling < 40. All findings were negative per CDC standards. A Cunningham Panel™ was repeated (Test 2) to assess the post-treatment status of antineuronal antibodies. The results of the five assays were all within normal ranges (27) (see Table 2).

**Table 2:** Cunningham Panel results Post-treatment—test 2.

	**Dopamine D1 (titer)**	**Dopamine D2 (titer)**	**Lysoganglioside (titer)**	**Tubulin (titer)**	**CaM Kinase II (% of baseline)**
Patient result	1,000	8,000	40	500	108
Normal ranges	500–2,000	2,000–8,000	80–320	250–1,000	53–130
Normal mean	1,056	6,000	147	609	95
Interpretation	Normal	Borderline	Normal	Normal	Normal

At this point, it was determined that no further IVIg was needed. Because the family was unable to obtain insurance coverage for the IVIg treatments, they incurred the expense out-of-pocket which averaged $12,000 per treatment (33, 34). At her follow up visit 6 months later, 31 months after her initial visit, the patient's Lyme disease checklist indicated overall improvement. Her final laboratory testing for tick-borne diseases, strep antibodies, and *M. pneumoniae* were all within normal limits.

### Outcome of Treatment

Currently this patient appears to be fully recovered and has been discharged from the care of the pediatric Lyme disease specialist. She is asymptomatic and performing academically at the “top” of her class according to her mother. A summary of the serological evidence of exposure to tick borne illness and streptococcal infection is included in Table 3.

**Table 3:** Serological evidence of exposure to *B. burgdorferi, B. henselae, B. duncani* and streptococcal infections.

**Serological evidence of exposure to** ***Borrelia burgdorferi***	
Testing 1	Lyme ELISA +1.25 (0.00-0.94); Lyme WB IgM + 23, 41
Testing 2	Lyme WB IgM + 31, 41
Testing 3	Lyme WB IgM + 31, 41 and 58
**Serologic evidence of exposure to** ***B. henselae***	
*B. henselae* IgM neg; *B. henselae* IgG + 1:64 (<1:64)	
**Serologic evidence of exposure to** ***B. duncani***	
*B. duncani* IgM 80 (IgM >40 indicates active infection)	
**Serologic evidence of exposure to group A streptococcus**	
DNASE B + 407 (<376 u/mL)	

## Discussion

This patient's case may be representative of numerous other cases of autoimmune neuropsychiatric illnesses where a patient may have concomitant infections and co-morbid diagnoses. What is known with growing certainty is that post-infectious neuropsychiatric illness appears to be increasing in frequency or, at least, in frequency of diagnosis. In cases such as the one presented here, it can be challenging for a patient to deal with controversy surrounding the diagnosis of PANDAS and the legitimacy of a diagnosis of Lyme disease with a neuropsychiatric presentation. Patients with chronic neuropsychiatric symptoms who do not respond adequately to traditional psychotropic medications may have an underlying immune-mediated condition triggered by one or more infections as evidenced in this case report. In addition, patients with genetic susceptibility to immune dysregulation, such as identified in this patient, carriers of the *HLA-DRB1, 2*, and *4* genes may increase the likelihood of an autoimmune encephalopathy indicated by the presence of antineuronal antibodies associated with neuropsychiatric symptoms. The *HLA-DRB1* gene plays a critical role in the immune system, where the HLA complex helps the immune system distinguish the body's own proteins from proteins made by foreign invaders such as viruses and bacteria. There is increasing evidence that IVIg and immunoglobulins are effective in treating autoimmune neuropsychiatric illness although the mechanism of action is uncertain.

It is often noted that patients diagnosed with PANDAS or PANS are frequently found to have multiple co-infections (35). Technically, since the initial treatment of the strep with amoxicillin did not initially result in improvement in the patient's neuropsychiatric symptoms, whereas with the addition of clindamycin the patient's symptoms began to improve, it could be concluded that Lyme may have been the pathogenic agent. However, in clinical experience it has been found that improvement in neuropsychiatric symptoms does not typically occur unless all co-infections are addressed and resolved. Another explanation may be that once the co-offending infection was also resolved, the patient's neuropsychiatric symptoms began to improve. This also brings up an issue with nomenclature; since this patient had a Lyme infection, she could be clinically diagnosed with PANS, or because of the strep diagnosis, clinically this patient could also be diagnosed with PANDAS. Because this patient had concurrent infections, we have referred to this case as a patient with PANDAS and Lyme co-infection. We suggest that the utilization of the term “Basal Ganglia Encephalitis (BGE)” may have more clinical utility when referring to the possible pathophysiology, where the type of infectious trigger may not be essential in the nomenclature of the clinical syndrome (26, 36).

## Conclusion

The subject of this case report had a concomitant diagnosis of Lyme borreliosis and PANDAS, both of which are consistent with the neuropsychiatric symptoms she experienced. As evidenced by her recovery and resolution of symptoms, treating both the Lyme infection and streptococcal infection, as well as treating the underlying autoimmune etiology of her neuropsychiatric symptoms resulted in a successful outcome. This case report and treatment history reiterates the complex and challenging nature of infection-triggered autoimmune neuropsychiatric disorders such as PANDAS and PANS and that multiple concomitant infectious agents can frequently be identified in patients suffering from these complex neuropsychiatric disorders. The presence of elevated antineuronal antibodies identified by the Cunningham Panel™ provided an aid in the diagnosis and in directing immunomodulatory treatment. The post-treatment resolution of these autoantibodies provided pathophysiological support for addressing both the infection(s) and the underlying immune system dysfunction which resulted in a positive medical outcome for this patient.

## Data Availability Statement

The datasets generated for this study are available on request to the corresponding author.

## Ethics Statement

Written informed consent from the patient's mother was obtained allowing the submission and publication of this case study.

## Author Contributions

CJ: treating physician of subject of case study, provided oversight, and approval of manuscript. CS: provided oversight and technical assistance during the preparation of the manuscript. AC: provided case review and draft development of the manuscript. DB: treating physician and provided case review. All authors contributed to the article and approved the submitted version.

## Conflict of Interest

AC and CS are employed by Moleculera Labs. The remaining authors declare that the research was conducted in the absence of any commercial or financial relationships that could be construed as a potential conflict of interest.
